# Multilevel unilateral versus bilateral pedicular percutaneous vertebroplasty for osteoporotic vertebral compression fractures

**DOI:** 10.3389/fsurg.2022.1051626

**Published:** 2023-01-06

**Authors:** Yixuan Tan, Jia Liu, Xiaoming Li, Liangqin Fang, Duowen He, Junming Tan, Guohua Xu, Xuhui Zhou

**Affiliations:** ^1^ Department of Orthopedics, Spine Center, Second Affiliated Hospital of Naval Medical University, Shanghai, China; ^2^ Department of Orthopedics, The 72nd Army Hospital of the People’s Liberation Army, Huzhou, China; ^3^ Department of Organ Transplant Center, Second Affiliated Hospital of Naval Medical University, Shanghai, China

**Keywords:** vertebroplasty, bilateral, unilateral, multiple levels, vertebral compression fracture

## Abstract

**Study design:**

Retrospective study.

**Objective:**

Controversy exists over the need for unilateral vs. bilateral pedicular percutaneous vertebroplasty (PVP) for patients with osteoporotic vertebral compression fractures (OVCFs). Clinical research is scarce comparing two approaches for multi-level PVP. This study aimed at evaluating the clinical and radiographic outcomes of multi-level PVP using two approaches for OVCFs.

**Methods:**

Seventy-eight patients with OVCFs undergoing multi-level PVP were enrolled including 36 patients undergoing unilateral PVP and 42 undergoing bilateral PVP. The clinical and radiological assessments including the Visual Analogue Scale (VAS), sagittal and coronal segmental Cobb, vertebral compression ratio (VCR), and vertebral sides ratio (VSR) were evaluated preoperatively and postoperatively.

**Results:**

All patients achieved a minimum 2-year follow-up. A total of 164 fractured vertebrae were enrolled. Regarding clinical efficacy, the VAS score improved in both groups after surgery, but the two groups did not differ significantly. The changes tendency in Sagittal Segmental Cobb, VSR, and VCR were similar postoperatively, and no statistically significant difference between groups. As for the Coronal Segmental Cobb angle, patients in unilateral and bilateral groups were shown to have 5.0° ± 4.0° and 2.6° ± 2.2° degrees loss of correction at a minimum 2-years follow-up duration, respectively. The loss of correction in the Coronal Segmental Cobb of unilateral group was significantly greater than that of bilateral group.

**Conclusion:**

Both multi-level unilateral and bilateral pedicular PVP achieved significant pain reduction and vertebral height restoration. Moreover, the bilateral PVP has shown advantages in stabilizing Coronal Cobb angle in patients with OVCFs.

## 1. Background

Osteoporotic vertebral compression fractures (OVCFs) in the thoracolumbar junction are common fractures caused by the progression of osteoporosis with high or low energy violent trauma. There is a lack of consensus on the best treatment and surgical indications for OVCFs (1, 2). The percutaneous vertebroplasty (PVP) and percutaneous kyphoplasty (PKP) treatment methods are widely accepted as the standard and effective techniques for treating OVCF in seniors. They demonstrated numerous advantages including height restoration, stabilization of the vertebral body, significant pain reduction, early mobilization, and significant mortality reduction (3).

The best choice of puncture routes for PVP raised much debate over the past decade (4). Recently, several articles compared the clinical and radiographic assessments of unilateral with bilateral vertebroplasty and suggested that both bilateral and unilateral percutaneous pedicular PVP achieved back pain alleviation and dysfunction improvement (3). However, most studies compared these two approaches for the treatment of patients with single-level PVP. To our knowledge, there is a paucity of the report focusing on multi-level PVP. In this study, we retrospectively evaluated and compared the clinical and radiographic outcomes of multilevel PVP utilizing unilateral and bilateral pedicular PVP at two institutes.

## 2. Materials and methods

### 2.1. Patient population

The retrospective clinical study has been approved by the Ethical Committee of two trauma centers (Grade III-A). Data from 363 patients diagnosed with OVCF at two grade III-A hospitals (spinal trauma centers) between January 2014 and April 2020 were retrospectively reviewed. From January 2014 to January 2016, we conducted the unilateral pedicular PVP, and from February 2016 to April 2020, we conducted the bilateral pedicular PVP. Therefore, the consecutive patients diagnosed with OVCFs were divided into two groups. Informed consent was obtained from each patient.

**The inclusion criteria were:** (1) The confirmatory diagnosis of vertebral compression fractures is made by a preoperative radiograph showing a wedge or biconcave morphology and magnetic resonance imaging (MRI) scan showing typical signal alteration within the fractured vertebrae; (2) Patients who underwent multi-level unilateral PVP (U-PVP) or bilateral PVP (B-PVP); (3) Patients with severe osteoporosis with the bone mineral density (BMD) *T*-score less than −2.5 standard deviation.

**The exclusion criteria were:** (1) Vertebral compression fractures with neurological deficit or with posterior columns destruction with bony fragment retropulsion; (2) Fractures that involved only one vertebra or treatment involved only single-level PVP; (3) The *T*-scores of patients do not meet the criteria of osteoporosis; (4) Obesity, severe cardiovascular diseases, or cancer.

From 363 reviewed cases, 285 patients were excluded owing to any of the exclusion criteria or incomplete radiographic and clinical data. 78 patients with multi-level OVCFs (23 males and 55 females, ages ranging from 52 to 85 years old) were enrolled in this study. They were all treated with the multi-level PVP, involving 36 patients who underwent unilateral pedicular percutaneous vertebroplasty (group U-PVP) and 42 who underwent bilateral pedicular percutaneous vertebroplasty (group B-PVP). The patients' general preoperative information is listed in Table 1 (no significant differences between the two groups were observed).

**Table 1:** Preoperative demographic data.

Variable	Un-PVP	Bi-PVP	*P*-value
Number^a^	36	42	-
Average age (year)^a^	72.0 ± 7.6	70.1 ± 7.1	0.260
Gender^b^			
Male	10	13	0.759
Female	26	29	
Number of PVP vertebrae^b^			
Total enrolled	36	42	-
2 Segments	32	39	0.831
>2 Segments	4	3	
Distribution of PVP vertebrae^b^			
Total enrolled	77	87	-
Thoracic vertebrae	26	31	0.802
Lumbar vertebrae	51	56	
Preoperative C-Cobb^a^	8.8 ± 4.4	7.7 ± 3.4	0.193
Preoperative S-Cobb^a^	22.8 ± 12.6	19.0 ± 8.8	0.133
Follow-up time (months)^a^	35.2 ± 14.0	34.1 ± 9.0	0.692

Data expressed as mean standard deviation unless otherwise indicated. C-Cobb, coronal segmental cobb; S-Cobb, sagittal segmental cobb.

^a^ Unpaired-samples *t*-test.

^b^ *χ*^2^ test.

### 2.2. Surgical methods

Using G-arm fluoroscopy, we determined body surface projections in the pedicle of fractured vertebras and marked them on the skin. Conventional disinfection and draping were performed. Briefly, patients were placed in the prone position and a soft pillow was placed under the two shoulders and anterior superior iliac spines. Procedures were performed under local anesthetic. The PVP procedure was performed using unilaterally or bilaterally as previously described under fluoroscopic guidance (5–7). Ideally, the puncture needles should be inserted and progressed into two-thirds of the anterior side of the vertebrae and the cement (polymethylmethacrylate, PMMA, Medtronic, Inc.) was injected incrementally to fill the vertebrae. Intraoperatively, the injection of the bone cement was examined using a G-arm *x*-ray. Subsequently, the skin was sutured with a non-absorbable suture. All patients resumed their regular activities the next day and received routine treatments of antiosteoporosis after surgery.

All procedures were performed by the same surgical team, with the same operator and assistant.

### 2.3. Evaluations

Patients' data including age, gender, symptoms, imaging results, management, follow-up periods, and clinical and radiographic outcomes were collected and analyzed (Table 1). All radiological measurements were performed independently by two researchers who were blinded to the operative approaches. Analysis was performed using the average values of the two observations at each level. Every patient was kept in a periodical follow-up for at least 2 years after surgery and the clinical and radiological assessments were listed below.

#### 2.3.1. Clinical evaluation

Clinical evaluation was assessed with the Visual Analogue Scale (VAS) preoperatively, 24 h, 3-months, and 12-months postoperatively.

#### 2.3.2. Radiographic evaluation

Radiological measurements were assessed before surgery, 24 h after surgery, and at the minimum follow-up duration of 2 years after the operation.

**Figure 1:**
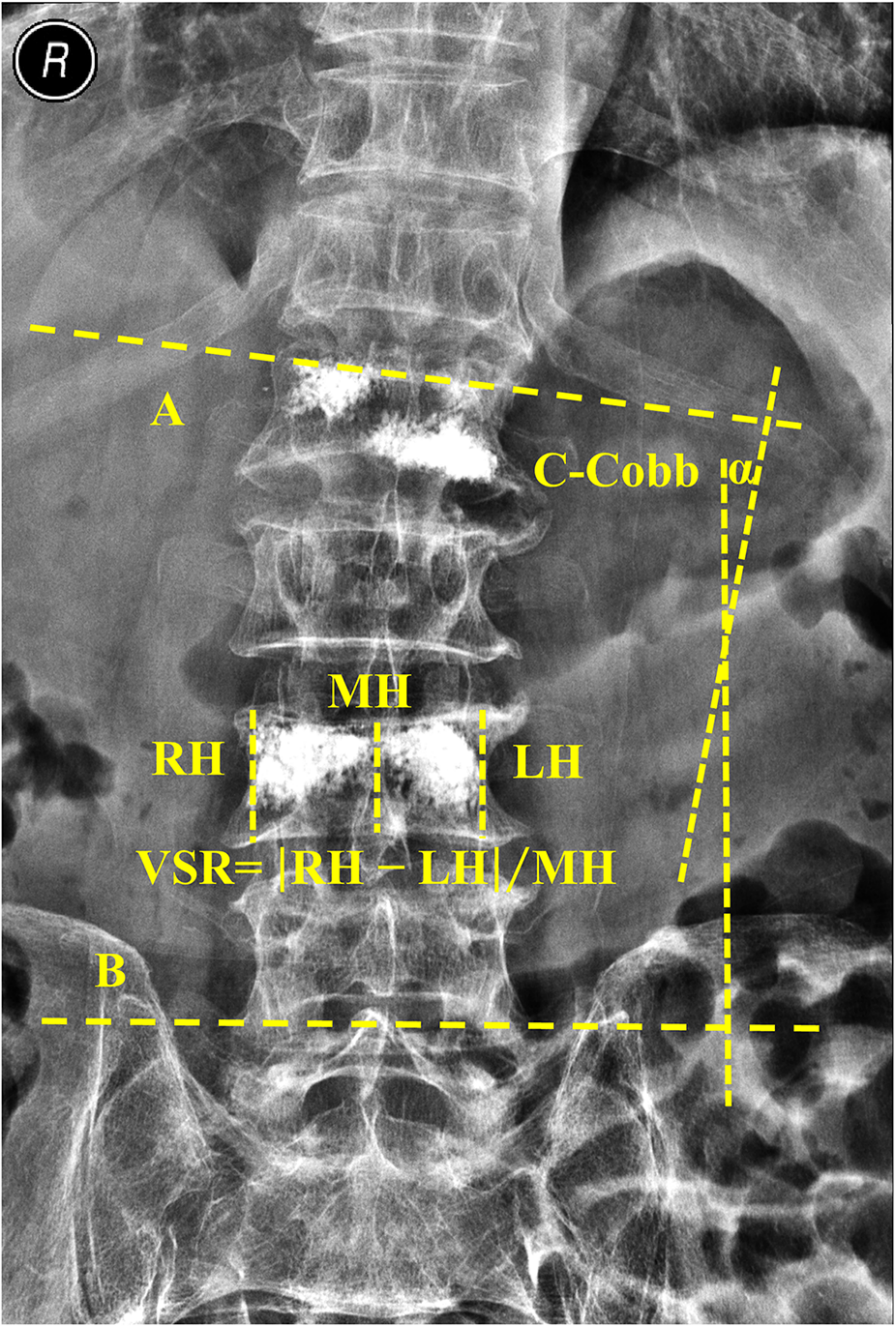
Measurements of coronal segmental cobb (C-cobb) and the vertebral side ratio (VSR). The heights of both left (LH), right (RH), and middle (MH) vertebral bodies were measured through an anteroposterior radiograph as shown in the Figure. The VSR was calculated as the following formula: VSR = |RH–LH|/MH. The Coronal Segmental Cobb (C-Cobb) angle α was measured from an anteroposterior radiograph by recording the angles between line A and line B, which were drawn parallel to the cranial and caudal most-tilted vertebral endplates.

**Figure 2:**
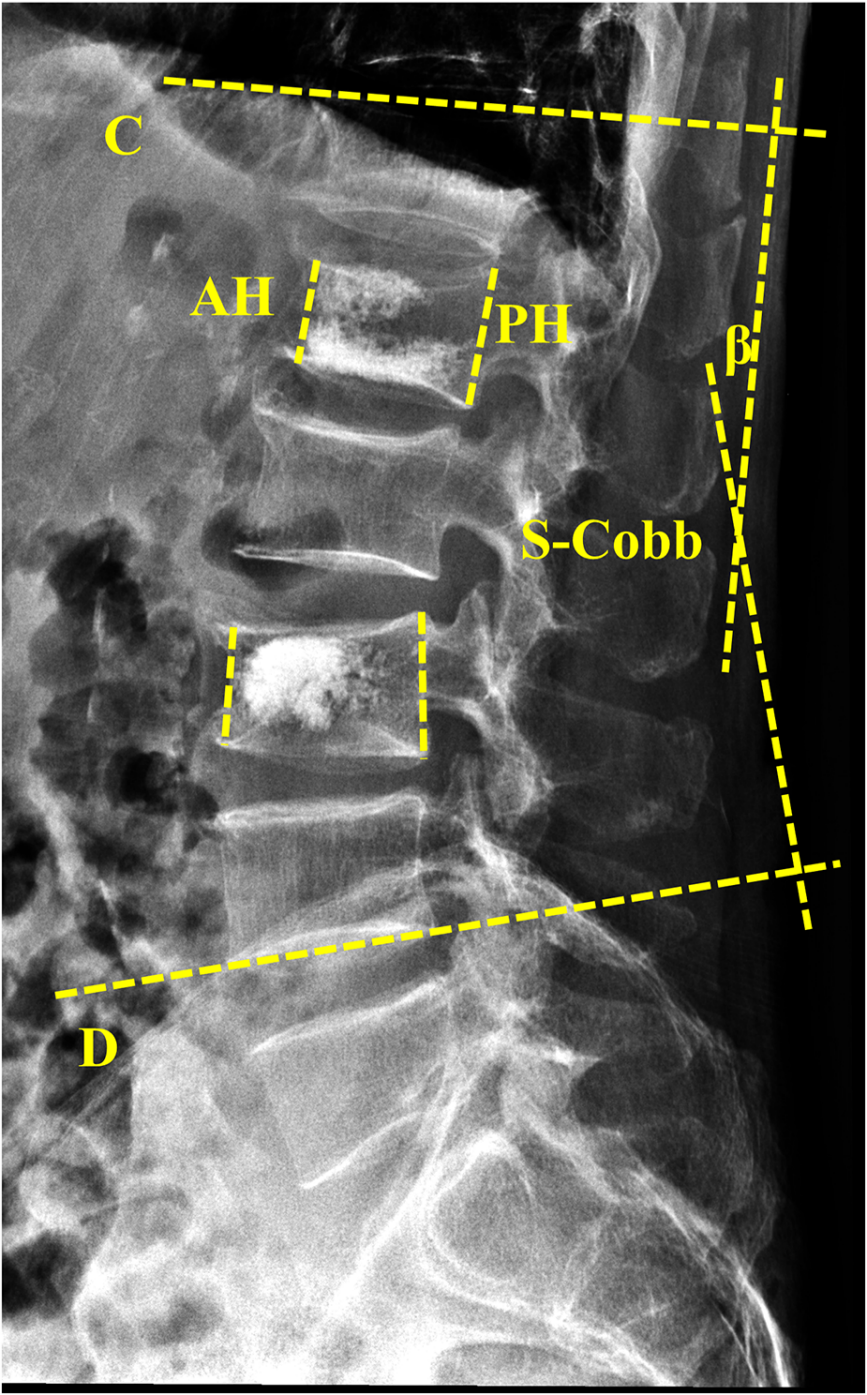
Measurements of sagittal segmental cobb (S-cobb) and the vertebral compression ratio (VCR). The heights of both anterior (AH) and posterior (PH) vertebral body were measured through lateral thoracolumbar radiography. The vertebral compression ratio (VCR) was calculated as the following formula: VCR = |PH–AH|/PH. The Sagittal Segmental Cobb (S-Cobb) angle β was measured from lateral thoracolumbar radiograph by recording the angles between line C and line D, which were measured between the inferior, superior adjacent treated vertebras through lateral radiographs.

The heights of both anterior (AH) and posterior (PH) vertebral body were measured through lateral thoracolumbar radiography, and the heights of both left (LH), right (RH), and middle (MH) vertebral body were measured through anteroposterior (AP) radiographs. The vertebral compression ratio (VCR) was calculated by using the following formula: VCR = [PH–AH]/PH (6). To further observe the morphology of the vertebral bodies, we introduced a new self-designed indicator, namely, vertebral side ratio (VSR). The vertebral side heights difference was calculated as the absolute difference of vertebral left and right heights, and then the ratio of the VSR was calculated as the ratio of vertebral side heights difference and middle vertebral height (Figure 1). Briefly, the VSR was calculated using the following formula: VSR = [RH–LH]/MH. The Coronal Cobb (C-Cobb) angle was measured from an anteroposterior radiograph by recording the angles between two lines, which were drawn parallel to the cranial and caudal most-tilted vertebral endplates (Figure 1). The measurement of the Sagittal Segmental Cobb (S-Cobb) angle was obtained between the inferior and superior adjacent treated vertebras through lateral radiographs (Figure 2) (6).

## 3. Statistical analysis

The SPSS statistical software (Version 25; IBM Corp., NY, United States) was used to complete the statistical analyses. Continuous variables were presented as mean with standard deviation, and categorical variables as total number and percentage. Continuous variables were compared using Student *t*-tests including patients' age, VCR, VSR, S-Cobb, and C-Cobb angle. Mann–Whitney *U* tests were used to compare VAS scores between the two groups. Comparisons of categorical variables including gender, fracture classification, and distribution of segments were performed with the *χ*^2^ test. The statistical significance was defined with a *P*-value < 0.05.

## 4. Results

**Figure 3:**
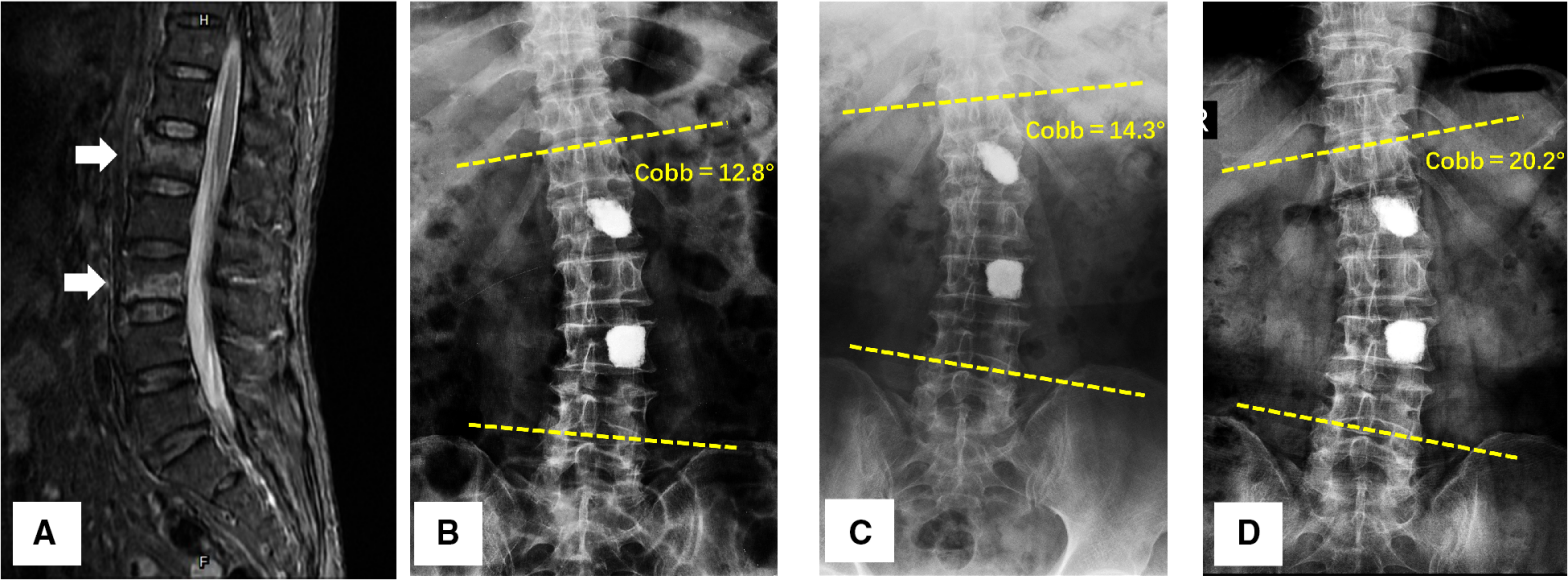
A 58-year-old female was diagnosed with L1 and L3 vertebral compressed fractures treated with unilateral PVP. (**A**) Preoperative T2-weighted MRI scan showed a higher-intensity signal within the fractured vertebral body; (**B–D**) 24 h after surgery, 6-month follow-up, and 48-month follow-up anteroposterior *x*-rays of lumbar spine, a progressive Coronal Segmental Cobb angle was observed with time.

**Figure 4:**
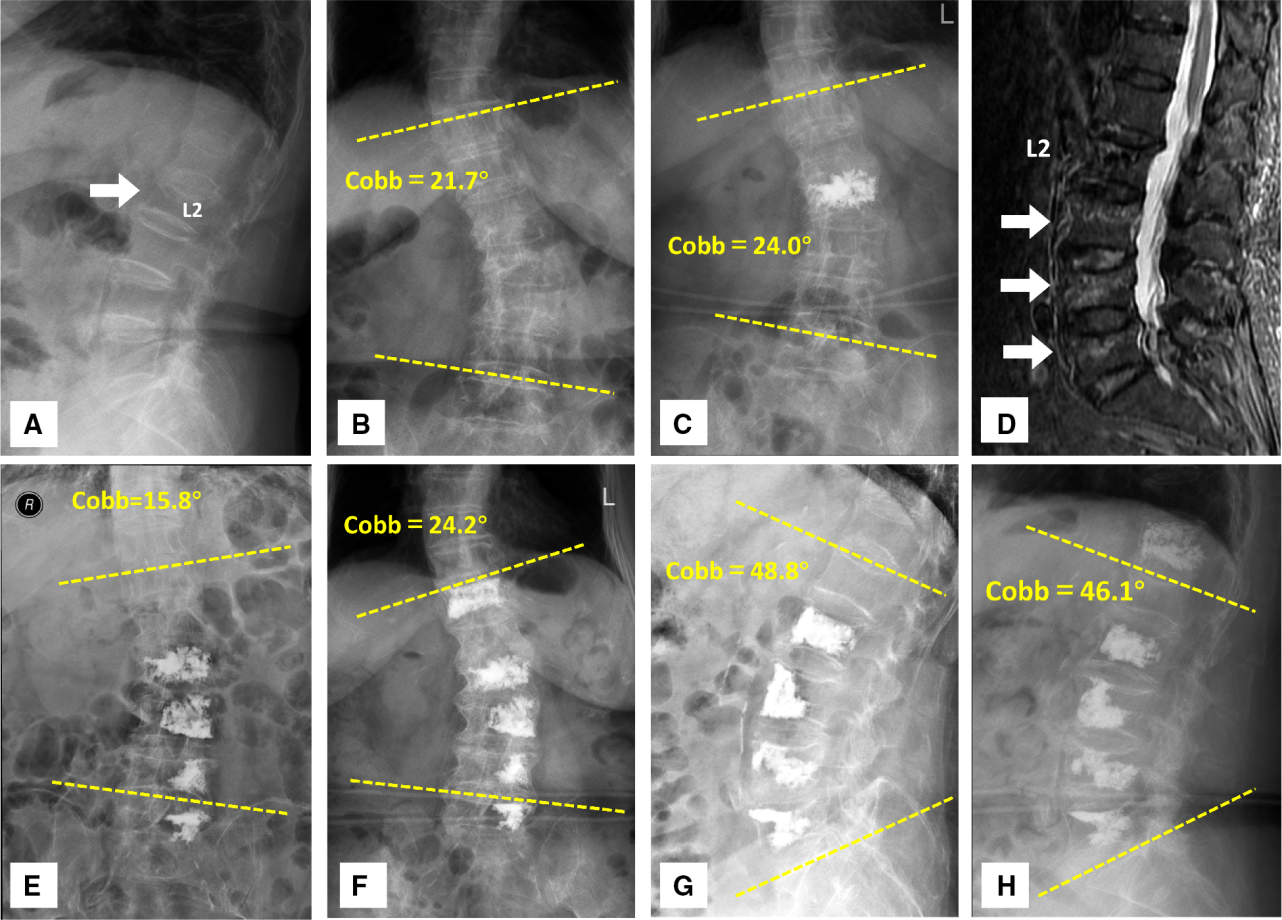
An 80-year-old woman presented with a history of osteoporotic vertebral compressive fractures and underwent multiple unilateral PVP. (**A**) The patient had been admitted for management of osteoporosis vertebral compressive fracture at L2 4 years ago. The white arrow indicated the fractured vertebra. (**B**) The preoperative radiograph showed scoliosis with a Cobb angle of 21.7°. (**C**) The patient was treated with PVP for L2 vertebra. The 24-h postoperative anteroposterior *x*-rays showed the cement augmentation within the L2 vertebra. (**D**) The patient was readmitted for management of progressive osteoporosis vertebral compressive fractures. MRI shows the signal alteration within the fractured vertebrae at L3, L4, and L5. The white arrows indicated the T2-weighted signal abnormalities in the fractured vertebrae. (**E,F**) A progressive Coronal Segmental Cobb angle was observed in the 24-h postoperative and 42-month follow-up anteroposterior *x*-rays of the thoracolumbar spine. A new T12 vertebral fracture had developed during the long-term follow-up and bilateral PVP were performed for the patient. (**G,H**) The postoperative and follow-up thoracolumbar *x*-rays demonstrated the mild changes in the sagittal segmental cobb angle.

**Figure 5:**
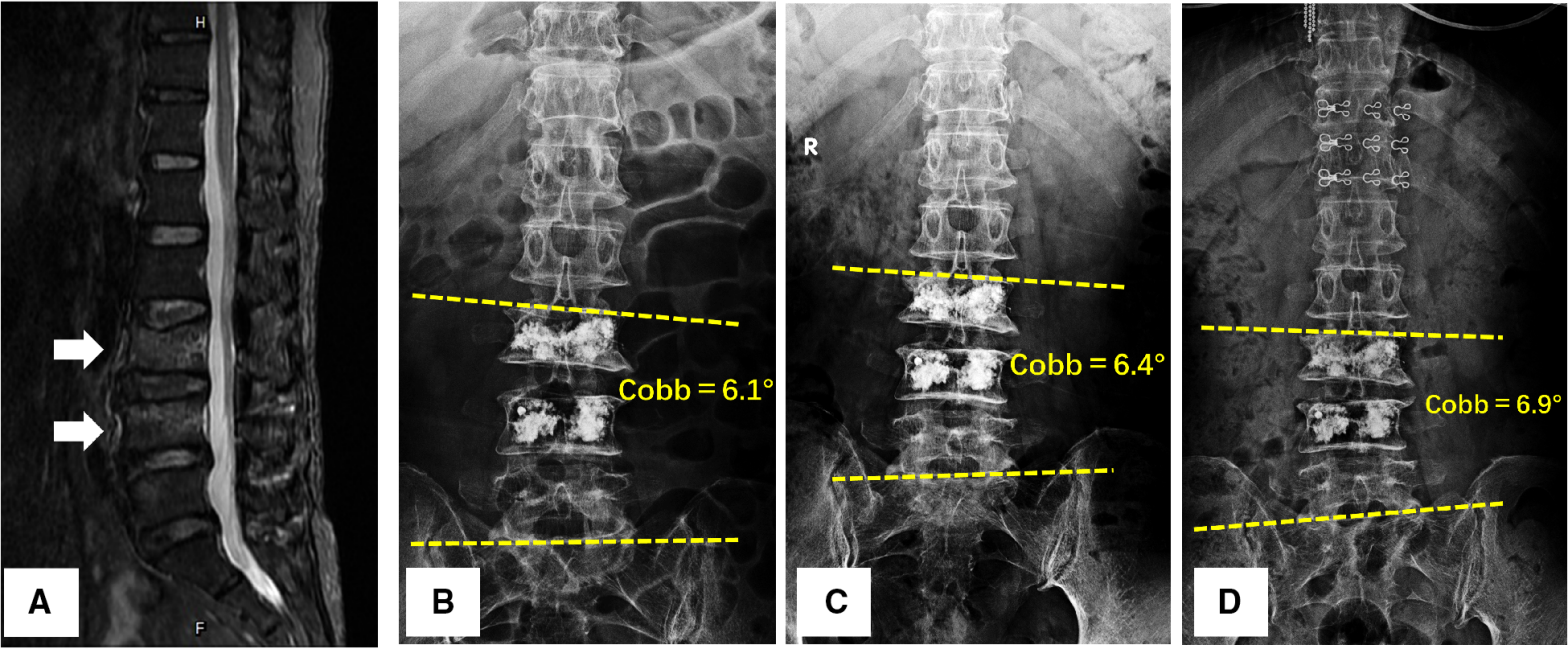
A 64-year-old female was diagnosed with L3 and L4 vertebral compressed fractures treated with bilateral PVP. (**A**) Preoperative T2-weighted MRI scan showed a higher-intensity signal within the fractured vertebral bodies; (**B–D**) 24 h after surgery, 6-month follow-up, and 48-month follow-up anteroposterior *x*-rays of lumbar spine, coronal segmental cobb angle varied little over time.

Seventy-eight patients (23 males and 55 females, ages ranging from 52 to 85 years old) with multi-level OVCFs were enrolled in this study. They were all surgically treated with the multi-level PVP and were divided into two groups according to the puncture routes: the unilateral group (U-PVP, *n* = 36) was treated with unilateral pedicular PVP and the bilateral group (B-PVP, *n* = 42) was treated with bilateral pedicular PVP. A total of 164 fractured vertebrae were enrolled including 77 vertebrae in the unilateral group and 87 vertebrae in the bilateral group. The distribution of involved vertebrae was as follows: T10: 6, T11: 23, T12: 28, L1: 36, L2: 31, L3: 17, L4: 13, L5: 10. All patients achieved a minimum two-year follow-up duration (average 34.6 months, ranging from 24 to 96 months). All patients' general preoperative information is listed in Table 1. There was no significant difference in general information. The typical cases are shown in Figures 3–5.

### 4.1. Clinical outcomes

No infections, vascular injuries, neurological injuries, cement pulmonary embolism, or any other serious complications was identified during or after operations in the two approaches. Bone cement leakage developed in 5 cases (13.9%) in the unilateral PVP and 7 cases (16.7%) in the bilateral PVP, but none of the patients had any serious neurologic symptoms or other significant clinical sequelae. The graph of the VAS pain score is shown in Table 2. The mean preoperative VAS scores were 7.4 ± 0.6 and 7.1 ± 1.0 points in U-PVP and B-PVP, respectively; One day after surgeries, all patients in both groups achieved significant pain relief, and the VAS scores decreased significantly to 3.2 ± 0.9 and 3.3 ± 1.0 in U-PVP and B-PVP, respectively (Mann–Whitney *U* tests, *Z* = −0.264, *P *= 0.791), but two approaches did not differ significantly. At the time of the 3 months after the operation, the VAS scores were 3.0 ± 0.7 and 3.1 ± 0.9, respectively, and reduced to 2.7 ± 0.6 and 2.8 ± 0.6 respectively at the time of the 12 months after the operation, no significant difference between the groups (Mann–Whitney *U* tests, *Z* = −0.315, *P *= 0.753).

**Table 2:** The changes of VAS scores during follow-up period.

Characteristic	Preoperative	1-Day post-op	3-Month post-op	12-Month post-op
U-PVP	7.4 ± 0.6	3.2 ± 0.9	3.0 ± 0.7	2.7 ± 0.6
B-PVP	7.1 ± 1.0	3.3 ± 1.0	3.1 ± 0.9	2.8 ± 0.6
*P*-value	0.220	0.791	0.814	0.753

U-PVP, unilateral PVP; B-PVP, bilateral PVP; post-op, post-operative follow-up. The between-group comparisons were tested using Mann–Whitney *U* test.

### 4.2. Radiographic outcomes

Preoperative and postoperative radiological assessments of two groups were measured and listed in Tables 3–5. The preoperative Sagittal Segmental Cobb (S-Cobb) angle was 22.8° ± 12.6° and 19.0° ± 8.8° in U-PVP and B-PVP, respectively. It reduced significantly to 17.8° ± 11.5° and 15.1° ± 7.4° after the surgery, with the mean reduction of 5.8° ± 3.6° and 4.7° ± 3.8°. Significant reductions were recorded, but the two approaches did not differ significantly (*t*-test, *F* = 0.086, *P *= 0.228). All patients achieved a minimum 2-year follow-up. At the last follow-up, the S-Cobb increased from 17.8° ± 11.5° to 24.4° ± 10.8° in U-PVP, and from 15.1° ± 7.4° to 16.5° ± 10.1° in B-PVP. There was an 8.0° ± 5.6° loss of correction in the S-Cobb angle at the last visit for U-PVP and 6.9° ± 5.2° degrees for B-PVP. No significant difference was found between the two approaches in loss of correction in S-Cobb (unpaired-samples *t*-test, *F* = 0.113*, P *= 0.349) (Table 3).

**Table 3:** Preoperative and postoperative sagittal segmental cobb (S-Cobb) angle and loss of correction during the follow-up.

Parameter	U-PVP (*n* = 36)	B-PVP (*n* = 42)	*P*-value
Preoperative (°)	22.8 ± 12.6	19.0 ± 8.8	0.133
Regained after PVP (°)	5.8 ± 3.6	4.7 ± 3.8	0.228
Loss of correction (°)	8.0 ± 5.6	6.9 ± 5.2	0.349

*P*, *P* value; The between-group comparisons were tested using unpaired-samples *t*-tests.

**Table 4:** Preoperative and postoperative coronal cobb (C-Cobb) angle and loss of correction during the follow-up.

Parameter	U-PVP (*n* = 36)	B-PVP (*n* = 42)	*P-*value
Preoperative (°)	8.8 ± 4.4	7.7 ± 3.4	0.193
Regained after PVP (°)	3.1 ± 1.8	2.8 ± 2.2	0.487
Loss of correction (°)	5.0 ± 4.0	2.6 ± 2.2	0.03*

*P*, *P* value; The between-group comparisons were tested using unpaired-samples *t*-test.

* The symbol indicates significant difference between groups.

**Table 5:** The changes of vertebral sides ratio and vertebral compression ratio during follow-up period.

Parameter	Pre-operation	Post-operation (24 h)	Last follow-up
Group	U-PVP (*n* = 77)	B-PVP (*n* = 87)	*P*-value	U-PVP (*n* = 77)	B-PVP (*n* = 87)	*P*-value	U-PVP (*n* = 77)	B-PVP (*n* = 87)	*P*-value
VSR (%)	10.1 ± 6.7	11.2 ± 10.6	0.431	9.3 ± 6.9	8.3 ± 8.2	0.406	14.0 ± 12.8	12.1 ± 11.5	0.319
VCR (%)	20.5 ± 8.4	18.7 ± 8.7	0.169	10.2 ± 8.2	11.3 ± 7.5	0.400	17.0 ± 8.6	15.3 ± 8.5	0.208

VSR, vertebral sides ratio; VCR, vertebral compression ratio. The between-group comparisons were tested using unpaired-samples *t*-test.

As for the Coronal Segmental Cobb (C-Cobb) angle, the preoperative C-Cobb angle was 8.8° ± 4.4° and 7.7° ± 3.4° in U-PVP and B-PVP, respectively. At the last follow-up, the C-Cobb increased to 12.1° ± 4.0° in U-PVP, and 7.9° ± 3.3° in B-PVP. Patients in the U-PVP and B-PVP were shown to have 5.0° ± 4.0° degrees and 2.6° ± 2.2° degree loss of correction compared with that of 24 h after surgery. The loss of correction in the C-Cobb of U-PVP was significantly greater than that of B-PVP (unpaired-samples *t*-test, *F* = 13.372, *P *= 0.03) (Table 4).

In the U-PVP, the average vertebral compression ratio (VCR) was restored from 20.5 ± 8.4% preoperatively to 10.2 ± 8.2% postoperatively and increased to 17.0 ± 8.6% at the last follow-up. As for B-PVP, the corresponding percentages were 18.7 ± 8.7%, 11.3 ± 7.5%, and 15.3 ± 8.5%. The two approaches did not differ significantly at three different times (unpaired-samples *t*-test, *F* = 0.188, 0.843, 0.014, *P *= 0.169, 0.400, 0.208, respectively) (Table 5).

The preoperative vertebral sides ratios (VSR) were 10.1 ± 6.7% and 11.2 ± 10.6% in U-PVP and B-PVP, respectively. The postoperative VSR in the U-PVP changed from 9.3 ± 6.9% to 14.0 ± 12.8% during at least two years of follow-up periods. As for B-PVP, the corresponding ratios were 8.3 ± 8.2% and 12.1 ± 11.5%. No significant difference was found between the two approaches in value change of VSR at three different times (unpaired-samples *t*-test, *F* = 10.533, 3.129, 0.551, *P *= 0.431, 0.406, 0.319, respectively) (Table 5).

## 5. Discussion

Percutaneous vertebroplasty (PVP) is a safe and effective means of treatment for OVCF patients suffering from acute or refractory chronic pain compared with conservative management (8). Furthermore, previous studies reported that PVP performed at a single fracture level or that performed at multiple fracture levels were equally effective and consistent in the reduction of pain and the improvement in functional status (9, 10). However, some studies argued that the patients treated with single-level PVP have better clinical outcomes than multi-level PVP patients owing to factors such as decreased local kyphosis (6, 11). In the present study, we also found both the treatments of unilateral and bilateral PVP could achieve a good therapeutic effect for the patients with multi-level fractures, though we have not compared them with the outcomes of single-level fractures.

Is unilateral PVP or bilateral PVP more effective for OVCF? The choice of puncture routes for PVP raised much debate regarding its clinical efficacy over the past decade. Theoretically, unilateral pedicular PVP can shorten the operation time and reduce *x*-ray exposure, hence some reported that unilateral PVP was superior to bilateral PVP in terms of shortening the operation time and reduction of operative complications (3, 12). However, some current studies showed that unilateral and bilateral pedicular PVP can achieve equivalent radiographic and clinical outcomes. They reported that unilateral PVP is comparable to the bilateral approach in the significant pain reduction, back dysfunction improvement, and restoration of vertebral body strength (4, 7, 13–16). Tohmen et al. conducted biomechanical tests on cadavers and found that unilateral and bilateral kyphoplasty had no significant difference in restoring vertebral bodies stiffness and strength (4, 13).

Despite the number of studies comparing the efficacy of single-level OVCF using unilateral with bilateral vertebroplasty, studies focusing on multi-level PVP have been rare. In the present study, long-term follow-up assessments were conducted to retrospectively compare the two approaches for the multilevel OVCF. We found that both unilateral and bilateral pedicular PVP led to significant relief of pain and height restoration of vertebrae. S-Cobb and C-Cobb have been used as parameters to describe the thoracolumbar sagittal and coronal balance. Though no significant difference was observed between groups in terms of sagittal balance, the loss of correction in the C-Cobb of the bilateral approach was significantly lower than that of the unilateral approach, which indicated that the bilateral PVP has advantages in stabilizing coronal balance for patients with OVCFs. Furthermore, this difference was not due to variation of single fractured vertebral body heights, which has been found by no significant difference between the two groups in value change of VSR. We believed that the biomechanical balance involved not only the fractured segments but also the adjacent segment and intervertebral disc. All these factors play an important role in preventing secondary scoliosis after surgery.

The cement distribution affects biomechanical balance (17). Symmetric placement of bone cement within the vertebral body during the PVP procedure is recommended (4, 18). For bilateral PVP, there was a more symmetric increase in stiffness on both sides of the vertebrae following cement augmentation. However, for unilateral PVP, the cement augmentation was restricted to one side of the vertebra body, and only that side's stiffness significantly increased. Consequently, the stiffness of the non-augmented side would remain significantly lower than that of the augmented side, leading to a biomechanical imbalance of stress on the vertebrae and adjacent vertebrae (18, 19).

In the present study, enrolled patients had primary or secondary osteoporosis. Multi-level PMMA applied axial load to the lumbar vertebral bodies and intervertebral discs, which significantly affected the biomechanical behavior of the thoracolumbar junction which is the region with the greatest load and mobility in the spine. We took into consideration the effect of the segmental distribution of fractured vertebrae in this study. According to a previous study, PKP was able to achieve better kyphosis correction in thoracic versus lumbar OVCF, and the sagittal alignment was also better maintained in the thoracic spine (11). Hence, we have compared the segmental distribution in the two groups and found no significant differences.

To make the cement distribution more symmetrically, some studies have proposed a unilateral extra-pedicular approach and a transverse process root-pedicle approach (5). however, these approaches may increase the risk such as difficulty puncturing, cement leakage, and pedicle fractures. In our opinion, it is not so easy for the unilateral approach to inject the bone filler to the opposite side and achieve asymmetric cement distributions.

The most common intraoperative complication for PVP was cement leakage, pedicle fracture, and medial transgression. In the presented study, no differences were found in complications between the two groups. However, for bilateral PVP, once PMMA leakage occurred on one side during the surgery, the injection on the other side can compensate for the inadequacy of cement due to the extravasation of PMMA outside the vertebral body. In clinical work, we found that the amount of bone cement injection in bilateral PVP is higher than that in unilateral PVP, which was consistent with the previous study (6, 20). Though only a small amount of bone cement is needed to achieve the effect of pain relief and restore the vertebral stiffness (18, 21). The sufficient cement augmentation contacting both upper and lower endplates can better restore the vertebral body's strength, reduce the risk of the vertebral body recompression, and maintain the height of the vertebral body (22). Moreover, with the application of G-arm *x*-ray machine and improvement of PMMA properties in clinical practice, there is decreasing difference between bilateral and unilateral PVP in terms of the amount of radiation exposure and the operation time, which is also the reason why bilateral PVP is regarded as the mainstay application of percutaneous vertebroplasty for the treatment of OVCFs in clinical practice.

There are some limitations to our study. This was a retrospective cohort study, so retrospective bias exists in the statistics. Further randomized controlled studies with a larger sample size should be conducted to verify the current result. Secondly, our research and conclusions relied highly on the data from our manual measurement. The bias in manual measurement may influence the radiographic values and outcomes. Thirdly, we did not evaluate the influence factor of bone cement distribution in the two approaches through computed tomography scanning. The location of cement within the vertebrae following injection is an important factor in determining the vertebral biomechanical balance, especially for the multiple fractures treated with multilevel PVP. Moreover, we have not compared the outcomes of multilevel PVP with single-level PVP, so our conclusions may not be generalizable to all patients.

## 6. Conclusion

Both multi-level unilateral and bilateral pedicular PVP achieved significant pain reduction and vertebral height restoration. However, from a radiographic perspective in the long-term follow-up, the bilateral PVP has shown advantages in stabilizing Coronal Cobb angle in patients with multi-level OVCFs compared to the unilateral approach. Hence, in the treatment of multiple-level OVCFs, we still recommend the use of the bilateral pedicular PVP as the preferred surgical technique.

## Data Availability

The original contributions presented in the study are included in the article/Supplementary Material, further inquiries can be directed to the corresponding author.

## References

[1] Diniz JM Botelho RV. Is fusion necessary for thoracolumbar burst fracture treated with spinal fixation? A systematic review and meta-analysis. J Neurosurg Spine. (2017) 27(5):584–92. 10.3171/2017.1 28777064

[2] Lan T Chen Y Hu SY Li AL Yang XJ. Is fusion superior to non-fusion for the treatment of thoracolumbar burst fracture? A systematic review and meta-analysis. J Orthop Sci. (2017) 22(5):828–33. 10.1016/j.jos.2017.05.014 28641907

[3] Yilmaz A Çakir M Yücetaş CŞ Urfali B Üçler N Altaş M Percutaneous kyphoplasty: is bilateral approach necessary? Spine. (2018) 43(14):977–83. 10.1097/brs.0000000000002531 29280933

[4] Steinmann J Tingey CT Cruz G Dai Q. Biomechanical comparison of unipedicular versus bipedicular kyphoplasty. Spine. (2005) 30(2):201–5. 10.1097/01.brs.0000150831.46856.87 15644756

[5] Zhang W Liu S Liu X Li X Wang L Wan Y. Unilateral percutaneous vertebroplasty for osteoporotic lumbar compression fractures: a comparative study between transverse process root-pedicle approach and conventional transpedicular approach. J Orthop Surg Res. (2021) 16(1):73. 10.1186/s13018-021-02219-6 33478545 PMC7818944

[6] Chen L Yang H Tang T. Unilateral versus bilateral balloon kyphoplasty for multilevel osteoporotic vertebral compression fractures: a prospective study. Spine. (2011) 36(7):534–40. 10.1097/BRS.0b013e3181f99d70 21242864

[7] Yan L He B Guo H Liu T Hao D. The prospective self-controlled study of unilateral transverse process-pedicle and bilateral puncture techniques in percutaneous kyphoplasty. Osteoporos Int. (2016) 27(5):1849–55. 10.1007/s00198-015-3430-5 26608054

[8] Klazen CA Lohle PN de Vries J Jansen FH Tielbeek AV Blonk MC Vertebroplasty versus conservative treatment in acute osteoporotic vertebral compression fractures (vertos II): an open-label randomised trial. Lancet. (2010) 376(9746):1085–92. 10.1016/S0140-6736(10)60954-3 20701962

[9] Singh AK Pilgram TK Gilula LA. Osteoporotic compression fractures: outcomes after single- versus multiple-level percutaneous vertebroplasty. Radiology. (2006) 238(1):211–20. 10.1148/radiol.2381042078 16373769

[10] Moulin B Tselikas L Gravel G Al Ahmar M Delpla A Yevich S Safety and efficacy of multilevel thoracolumbar vertebroplasty in the simultaneous treatment of six or more pathologic compression fractures. J Vasc Interv Radiol. (2020) 31(10):1683–1689.e1. 10.1016/j.jvir.2020.03.011 32921566

[11] Pradhan BB Bae HW Kropf MA Patel VV Delamarter RB. Kyphoplasty reduction of osteoporotic vertebral compression fractures: correction of local kyphosis versus overall sagittal alignment. Spine. (2006) 31(4):435–41. 10.1097/01.brs.0000200036.08679.1e 16481954

[12] Chen YC Zhang L Li EN Ding LX Zhang GA Hou Y Unilateral versus bilateral percutaneous vertebroplasty for osteoporotic vertebral compression fractures in elderly patients: a meta-analysis. Medicine. (2019) 98(8):e14317. 10.1097/MD.0000000000014317 30813133 PMC6408113

[13] Tohmeh AG Mathis JM Fenton DC Levine AM Belkoff SM. Biomechanical efficacy of unipedicular versus bipedicular vertebroplasty for the management of osteoporotic compression fractures. Spine. (1999) 24(17):1772–6. 10.1097/00007632-199909010-00004 10488505

[14] Papanastassiou ID Eleraky M Murtagh R Kokkalis ZT Gerochristou M Vrionis FD. Comparison of unilateral versus bilateral kyphoplasty in multiple myeloma patients and the importance of preoperative planning. Asian Spine J. (2014) 8(3):244–52. 10.4184/asj.2014.8.3.244 24967037 PMC4068843

[15] Rebolledo BJ Gladnick BP Unnanuntana A Nguyen JT Kepler CK Lane JM. Comparison of unipedicular and bipedicular balloon kyphoplasty for the treatment of osteoporotic vertebral compression fractures: a prospective randomised study. Bone Joint J. (2013) 95-B(3):401–6. 10.1302/0301-620X.95B3.29819 23450028

[16] Chen C Wei H Zhang W Gu Y Tang G Dong R Comparative study of kyphoplasty for chronic painful osteoporotic vertebral compression fractures via unipedicular versus bipedicular approach. J Spinal Disord Tech. (2011) 24(7):E62–65. 10.1097/BSD.0b013e318228f470 21822151

[17] Chen BL Li YQ Xie DH Yang XX Zheng ZM. Comparison of unipedicular and bipedicular kyphoplasty on the stiffness and biomechanical balance of compression fractured vertebrae. Eur Spine J. (2011) 20(8):1272–80. 10.1007/s00586-011-1744-3 21384203 PMC3175856

[18] Liebschner MA Rosenberg WS Keaveny TM. Effects of bone cement volume and distribution on vertebral stiffness after vertebroplasty. Spine. (2001) 26(14):1547–54. 10.1097/00007632-200107150-00009 11462084

[19] Li Y Cui W Zhou P, Li C, Wen Y, Xiao W. Comparison of a flexible versus rigid bone cement injection system in unilateral percutaneous vertebroplasty. Eur J Med Res. (2020) 25(1):36. 10.1186/s40001-020-00436-z 32843077 PMC7449043

[20] Chen Y Zhang H Chen H, Ou Z, Fu Y, Zhang J. Comparison of the effectiveness and safety of unilateral and bilateral percutaneous vertebroplasty for osteoporotic vertebral compression fractures: a protocol for systematic review and meta-analysis. Medicine. (2021) 100(51):e28453. 10.1097/MD.0000000000028453 34941201 PMC10545173

[21] Li Q Long X Wang Y, Guan T, Fang X, Guo D, et al. Clinical observation of two bone cement distribution modes after percutaneous vertebroplasty for osteoporotic vertebral compression fractures. BMC Musculoskel Dis. (2021) 22(1):577. 10.1186/s12891-021-04480-6 PMC8223328 34167517

[22] Tan L Wen B Guo Z Chen Z. The effect of bone cement distribution on the outcome of percutaneous vertebroplasty: a case cohort study. BMC Musculoskel Dis. (2020) 21(1):541. 10.1186/s12891-020-03568-9 PMC7427078 32791975

